# Barrier dysfunction or drainage reduction: differentiating causes of CSF protein increase

**DOI:** 10.1186/s12987-017-0063-4

**Published:** 2017-05-18

**Authors:** Mahdi Asgari, Diane A. de Zélicourt, Vartan Kurtcuoglu

**Affiliations:** 1 The Interface Group, Institute of Physiology, University of Zurich, Winterthurerstrasse 190, 8057 Zurich, Switzerland; 2 Neuroscience Center Zurich, University of Zurich, Zurich, Switzerland; 3 Zurich Center for Integrative Human Physiology, University of Zurich, Zurich, Switzerland

## Abstract

**Background:**

Cerebrospinal fluid (CSF) protein analysis is an important element in the diagnostic chain for various central nervous system (CNS) pathologies. Among multiple existing approaches to interpreting measured protein levels, the Reiber diagram is particularly robust with respect to physiologic inter-individual variability, as it uses multiple subject-specific anchoring values. Beyond reliable identification of abnormal protein levels, the Reiber diagram has the potential to elucidate their pathophysiologic origin. In particular, both reduction of CSF drainage from the cranio-spinal space as well as blood–CNS barrier dysfunction have been suggested ρas possible causes of increased concentration of blood-derived proteins. However, there is disagreement on which of the two is the true cause.

**Methods:**

We designed two computational models to investigate the mechanisms governing protein distribution in the spinal CSF. With a one-dimensional model, we evaluated the distribution of albumin and immunoglobulin G (IgG), accounting for protein transport rates across blood–CNS barriers, CSF dynamics (including both dispersion induced by CSF pulsations and advection by mean CSF flow) and CSF drainage. Dispersion coefficients were determined a priori by computing the axisymmetric three-dimensional CSF dynamics and solute transport in a representative segment of the spinal canal.

**Results:**

Our models reproduce the empirically determined hyperbolic relation between albumin and IgG quotients. They indicate that variation in CSF drainage would yield a linear rather than the expected hyperbolic profile. In contrast, modelled barrier dysfunction reproduces the experimentally observed relation.

**Conclusions:**

High levels of albumin identified in the Reiber diagram are more likely to originate from a barrier dysfunction than from a reduction in CSF drainage. Our in silico experiments further support the hypothesis of decreasing spinal CSF drainage in rostro-caudal direction and emphasize the physiological importance of pulsation-driven dispersion for the transport of large molecules in the CSF.

## Background

Despite continued advances in non-invasive medical imaging, cerebrospinal fluid (CSF) analysis in general and CSF protein analysis in particular have remained important tools for the diagnosis of various disorders of the central nervous system (CNS) [1]. Yet while it is accepted that abnormal changes in CSF protein content are indicative of pathological conditions, the reasons leading to the measured protein concentrations are often a matter of debate [2].

While some proteins found in the CSF are synthesized within the CNS (choroid plexus, brain and spine) or the meninges, most of them originate in the blood serum under normal conditions [2–4]. They pass through blood-CNS barriers (either the blood–brain barrier, BBB, or blood-CSF barrier, BCSFB) into CNS fluids [5]. Equilibrium between the rate-limited influx of serum derived proteins through these barriers and their efflux with CSF drainage determines the protein content of the CSF [6]. Changes in the concentrations of these proteins may thus reflect alterations in either (1) serum protein levels, (2) intrathecal protein synthesis [7], (3) barrier properties [8], or (4) CSF dynamics and drainage [2].

**Fig. 1:**
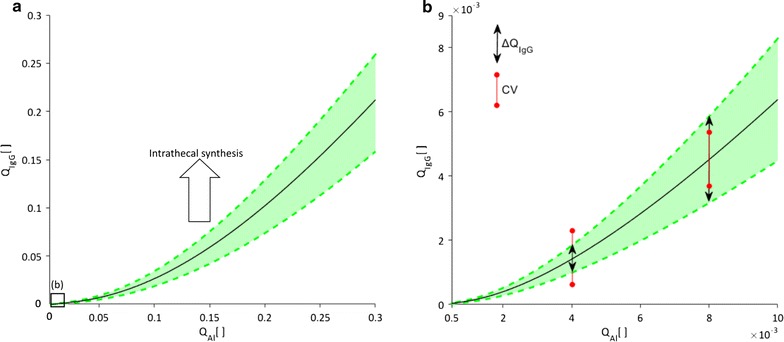
Variation of the immunoglobulin G quotient with that of albumin as depicted in Reiber diagrams. **a** Displays the empirically established relationship between the concentration of IgG in CSF relative to its concentration in blood serum (IgG quotient, Q_IgG_) and the correspondingly defined albumin quotient (Q_Al_). **b** Depicts the normal range of albumin quotients, corresponding to the area in **a** marked with the *black square*. Reiber demonstrated that the average quotient variation (*black line*) and upper and lower bounds (*green dashed lines*) follow the hyperbolic function $Q_{IgG} = \frac{a}{b}\sqrt {Q_{Al}^{2} + b^{2} } - c$ [32]. He also showed that the population variation coefficient (CV), defined for a given albumin quotient as $CV = \frac{{\Delta Q_{IgG} }}{{0.5\cdot\left( {Q_{{IgG_{upper limit} }} + Q_{{IgG_{lower limit} }} } \right)}}$, remains constant over the entire range of investigated albumin quotients. Q_IgG_ values above the upper bound are indicative of a blood-CNS barrier dysfunction

Since protein levels in the CSF show normal fluctuations as serum protein concentrations change, and since there are inter-individual variations, it is helpful to use relative values for diagnostic purposes. The Reiber diagram constitutes a standardized approach to assessing such values. Should, for example, the immunoglobulin G (IgG) concentration in a patient’s CSF sample be analyzed, its relative value with respect to serum IgG concentration (IgG quotient) is compared to the corresponding relative concentration of albumin (albumin quotient). Since albumin is not synthesized in the mature CNS [2], a higher than expected IgG quotient for the given albumin quotient is seen as evidence for intrathecal synthesis of IgG and thus for an inflammatory process in the CNS. When there is no intrathecal immunoglobulin synthesis, Reiber noted a hyperbolic relationship between immunoglobulin and albumin quotients as shown in Fig. 1, and stated that the albumin quotient should remain below 0.01 for normal subjects [2]. He further defined upper and lower bounds for the relationship between the two quotients, both of which also follow a hyperbolic function, and noted that the relative spread of these bounds, as quantified by a population variation coefficient, remains constant over the entire range of investigated albumin levels (Fig. 1b).

Of the four possible causes for changes in CSF protein concentration listed above, the Reiber diagram corrects for variations in serum protein levels and identifies intrathecal protein synthesis (see Fig. 1a). However, it cannot distinguish between changes in CNS barrier properties and changes in CSF dynamics and drainage, both of which have been hypothesized as possible causes for abnormal albumin quotients [2, 8, 9]. In this study, we have employed a set of computational tools to test these two competing hypotheses.

To this end, we have analysed how changes in barrier function, CSF drainage rates and pulsatility translate to changes of albumin and IgG quotients in the Reiber diagram, where IgG was chosen from the family of immunoglobulins arbitrarily as a common biomarker for inflammatory neurological disorders [10]. Our models reproduce the empirical mathematical relationship between the two quotients given by Reiber, quantify the effect of CSF pulsation on protein distribution and show that barrier dysfunction rather than decreased cerebrospinal fluid drainage is the likely cause of abnormally high albumin values in the Reiber diagram. Our results further emphasize the pathophysiological importance of dispersion, CSF drainage and blood-CNS barrier permeability for the transport of large molecules in the spinal subarachnoid space.

## Methods

**Fig. 2:**
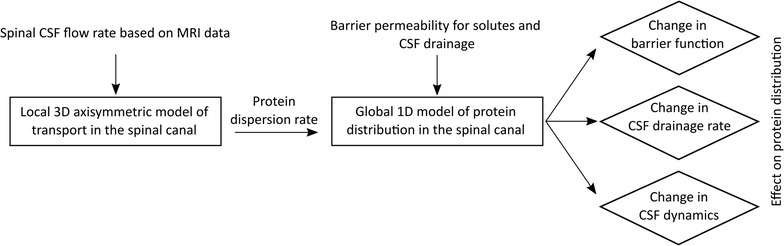
Study flow chart. This flow chart describes the application of the two computational models developed to test hypothesis about the cause of increased CSF albumin quotients. The modeling steps and hypotheses are framed by rectangles and rhombi, respectively, while model inputs and outputs are shown without bounding boxes

We designed two computational models (Fig. 2) to investigate the mechanisms governing protein distribution in the spinal CSF and underlying reasons for pathological changes in protein levels. With a one-dimensional model (presented second), we evaluate the distribution of albumin and IgG in the spinal CSF, accounting for the protein transport rate across blood-CNS barriers, CSF dynamics (including both dispersion induced by CSF pulsations and advection by mean CSF flow) and CSF drainage from the cranio-spinal space. We also study the impact of pathological changes in barrier permeability, CSF dynamics and drainage on these distributions. The dispersion coefficients used in this one-dimensional model to account for CSF pulsations are determined a priori by computing the axisymmetric three-dimensional CSF dynamics and solute transport in a representative segment of the spinal canal.

### Three-dimensional model of protein dispersion induced by CSF pulsation

Dispersion as the combined effect of diffusion and advection by pulsatile fluid motion with zero net flow is the governing mechanism for the faster transport of solutes in the CSF compared to pure diffusion [11–14]. To determine dispersion coefficients of albumin and IgG along the spine, we first solve the axisymmetric three-dimensional Navier–Stokes equations and associated advection–diffusion equation for protein transport in a segment of the spinal canal.

#### Model characteristics

**Fig. 3:**
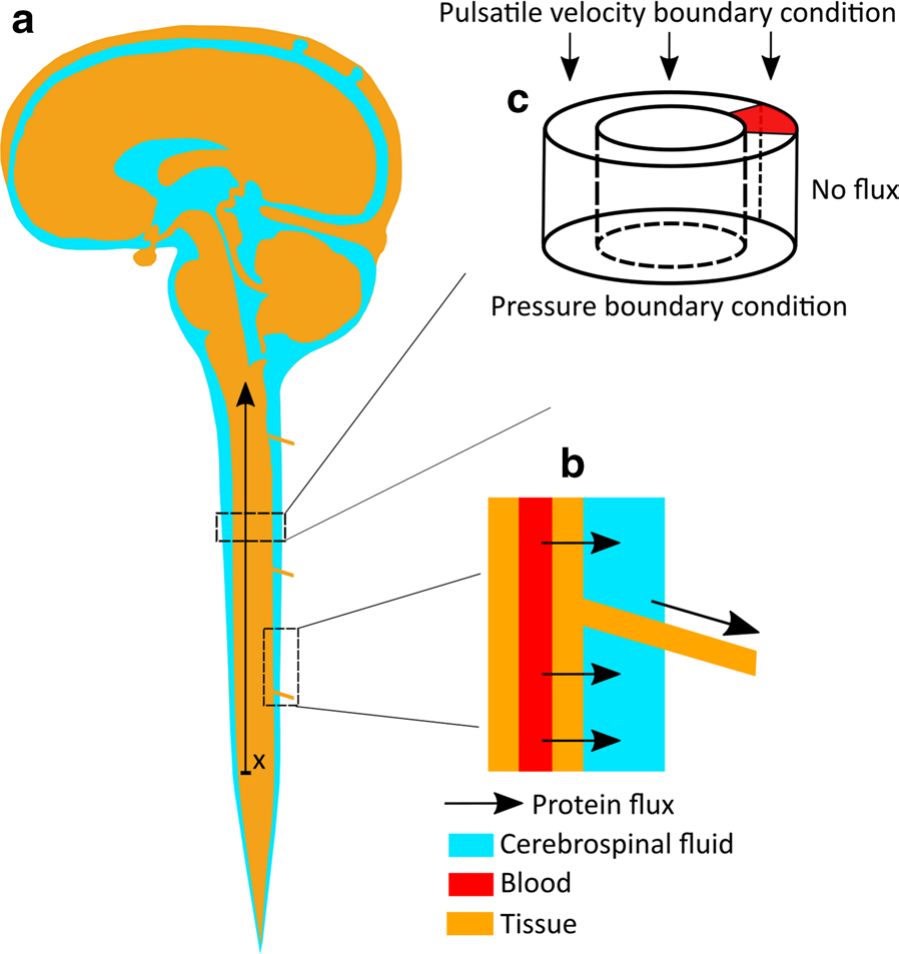
Schematic of the model domains. **a** A representation of the cerebrospinal fluid compartments. The *x* and *arrow* parallel to the spinal cord indicate the anatomic correspondence and orientation of the one-dimensional model. This orientation was chosen to match the direction in which CSF samples are accessed during sequential sampling of CSF through lumbar puncture [31]. **b** Protein efflux locations in the spine. Blood-derived proteins pass from blood by diffusion into the CSF space and exit it along nerve roots. **c** A representation of the three-dimensional model domain as an annular channel. The boundary conditions for this model are shown on the domain surfaces

The geometry of the spinal canal is idealized as an axisymmetric annular pipe (Fig. 3c) with dimensions based on statistical geometrical values reported in the literature [15, 16]. The thickness of the spinal subarachnoid space varies from cervical region to lumbar space within the range of 3.5–4.5 mm [17]. We have used the mean measured value for this thickness in the model, 4 mm [17]. The segment length is chosen to be long enough to avoid the influence of boundary conditions on protein transport rates. All geometrical parameters used are reported in Table 1.

**Table 1:** Model parameters

Parameter	Value	References
**Barrier permeability for albumin P**_**b**_ **[μg/min]**		
In the cortical subarachnoid space	29.4	[27]
In the ventricular space	7.6	[27]
In the spinal space	4.8	[27]
**CSF compartments volume [ml]**		
Ventricular space	30	
Cortical subarachnoid space	90	
Spinal subarachnoid space	30	
**Protein and pore size used in the membrane pore model for barrier permeability [nm]**		
Pore radius, r	19.4	[6]
Albumin hydrodynamic radius, $a_{Al}$	3.58	[6]
Immunoglobulin G hydrodynamic radius, $a_{IgG}$	5.34	[6]
**CSF production and drainage rate**		
CSF total production and drainage rate, F [ml/day]	500	[30]
CSF pulsation		
CSF pulsation amplitude in the cervical region [mm/s]	10	[25]
CSF pulsation amplitude in the lumbar region [mm/s]	0	[24]
CSF pulsation time period [s]	0.8	[25]
**CSF physical properties**		
Density, $\rho$ [kg/m^3^]	1000	
Viscosity, $\mu$ [Pa s]	0.001	
**Spinal canal porosity and permeability**		
Porosity, $\varepsilon$	0.99	[21]
Permeability in the longitudinal direction, K_longitudinal_ [m^2^]	1.45 · 10^−7^	[21]
Permeability in the radial direction, K_radial_ [m^2^]	2.36 · 10^−8^	[21]
**CSF albumin concentrations**		
Albumin concentration in the lumbar CSF [mg/ml]	0.363	[27]
Albumin CSF/blood quotient in the lumbar space	0.002	[31]
Albumin quotient ratio (lumbar to cisternal)	2	[27]
Albumin quotient ratio (cortical subarachnoid space to cisternal)	3	[27]
**Dimensions [mm]**		
Spinal cord diameter	10	[16, 17]
Spinal subarachnoid space thickness, w	4	[16, 17]
Spinal segment length	100	
Spine length between cistern and lumbar space	700	
Protein properties [m^2^/s]		
Albumin diffusion coefficient, D_Al_	6 · 10^−11^	
Immunoglobulin G diffusion coefficient, D_IgG_	2.4 · 10^−11^	

The model domain is treated as porous, with permeability and porosity metrics according to literature values for the subarachnoid space [18]. A velocity (flow) boundary condition derived from MRI measurements of spinal CSF [19] is imposed at the inlet boundary (proximal site), while a constant pressure boundary condition is imposed at the outlet (distal site). Both the inner and outer boundaries of the spinal canal are treated as impermeable walls with zero slip and zero solute flux conditions. Constant solute concentration is imposed at the axial boundaries.

#### Solution methodology

The time-dependent equations governing fluid motion and solute transport, namely modified Navier–Stokes with Darcy’s law for the porous medium, continuity and advection–diffusion equations, are solved numerically using the open source finite volume code OpenFOAM [20]:

1 $$\frac{{\partial {\text{u}}}}{{\partial {\text{t}}}} + \left( {{\text{u}}\cdot\nabla } \right){\text{u}} - \frac{\mu }{\rho }\nabla^{2} {\text{u}} = - \frac{1}{\rho }\nabla {\text{P}} - \frac{\mu \varepsilon }{{{\text{K}}\rho }}{\text{u}},$$

2 $$\nabla \cdot{\text{u}} = 0,$$

3 $$\frac{{\partial {\text{C}}}}{{\partial {\text{t}}}} = \left( {{\text{u}}\cdot\nabla } \right){\text{C}} + {\text{D}}\nabla^{2} {\text{C}},$$

where the unknowns u, P and C are, respectively, the fluid velocity, pressure, and protein concentration. The parameters µ and ρ are the dynamic viscosity and density of the cerebrospinal fluid, respectively, ε and K the porosity and permeability of the spinal canal, and D the diffusion coefficient of the respective protein. The permeability of the spinal subarachnoid space is derived using the solution presented by Gupta et al. [21]. The parameter values are reported in Table 1.

Equations (1) to (3) are discretized using an implicit Euler scheme for the temporal derivatives and central differencing for the first and second order spatial derivatives. All calculations are conducted with a time step size of 10^−4^ s and spatial resolution of 100 μm in both axial and radial directions. Grid and time-step independence were confirmed.

#### Evaluation of the dispersion coefficient

The dispersion coefficient may be derived from the above three-dimensional model by fitting the simulated axial concentration with the analytical solution of the dispersion equation in a semi-infinite domain [11]:

4 $$\frac{{{\text{C}}({\text{x}},{\text{t}})}}{{{\text{C}}_{0} ({\text{x}})}} = {\text{erfc}}\left( {\frac{\text{x}}{{2\sqrt {D_{L}^{*} {\text{t}}} }}} \right) ,$$

where x is the spatial coordinate in axial direction, t is time, ${\text{C}}_{0}$ is the initial concentration, and $D_{L}^{*}$ is the dispersion coefficient in a segment of length L. For a finite domain, this approximation is valid as long as the penetration Fourier number for the domain length remains small [22]. The value of $D_{L}^{*}$ is determined by fitting Eq. (4) to the results of the axisymmetric simulations at t = 8 s (10 cycles of pulsations). For further details on the dispersion coefficient evaluation, we refer the reader to [11].

### One dimensional model of protein distribution in the spinal CSF

Our one-dimensional domain represents protein transport in the spinal CSF between the lumbar and cervical regions. The model domain is illustrated in Fig. 3a. We solve the one-dimensional advection–diffusion equation modified to include sink and source terms representing protein drainage and influx, respectively, as schematically shown in Fig. 3b:

5 $$\frac{\partial C}{\partial t} = \frac{{\partial^{2} {\text{D}}^{ *} C}}{{\partial x^{2} }} + \frac{{\partial {\text{u}}C}}{\partial x} + S_{i} - S_{o} ,$$

where C(x,t) is the CSF protein concentration at time t and in axial location x, and u is the CSF bulk flow velocity. D* is the protein dispersion coefficient induced by CSF pulsation obtained from our three-dimensional model. The source term, S_i_, represents the influx of serum proteins into the CSF, while the sink term, S_o_, represents protein efflux due to CSF drainage [23]. The dimensions of the domain are reported in Table 1.

#### Evaluation of the dispersion coefficient D*

The dispersion coefficient depends on both the solute considered and the amplitude of the CSF pulsations. The latter has been shown to increase from zero in the lumbar space [24] to a maximum of about 10 mm/s in the cervical region [25]. Accordingly, we applied our three-dimensional model to characterize the dispersion coefficients of albumin and IgG for CSF pulsation amplitudes ranging between 0 and 10 mm/s. The corresponding dispersion values are reported in results section. Expectedly, dispersion equals to diffusion for the pulsation amplitude of zero (i.e. in the lumbar space) and increases for the higher pulsation amplitudes, reaching a maximum for 10 mm/s velocity (i.e. in the cervical space). Since there is an almost linear relation between the imposed velocity and calculated dispersion coefficient, we consider a linear increase of the dispersion coefficient from $D_{\hbox{min} }^{*}$ equal to the pure diffusion coefficient in the lumbar space to a value of $D_{\hbox{max} }^{*}$ in the cervical region.

#### Evaluation of the source term

In absence of active transporters in the blood vessel wall for albumin and immunoglobulins, the only transport mechanism for these larger proteins through the barrier is slow paracellular diffusion [26]. Therefore, the source term for the CSF concentration could be written as:

6 $$S_{i} = P_{b} \cdot(C_{blood} - C),$$

where P_b_ stands for the diffusive permeability of the blood-CNS barriers for the protein under consideration and $C_{blood}$ is the serum protein concentration. The permeability of the barrier to albumin molecules in different regions of the CSF compartments has been measured with radioactive studies [27]. However, it is not known how this permeability might change due to barrier opening. In order to model such permeability variations in pathological situations, we use the membrane pore model described in [6], which was demonstrated to accurately capture barrier permeability for different proteins. In this model, permeability depends on the ratio of protein size to pore size:

7 $$P_{b} \propto \left( {1 - \left( {a/r} \right)} \right)^{2} \cdot\left[ {1 - 2.1\cdot\left( {a/r} \right) + 2.09\cdot\left( {a/r} \right)^{3} - 0.95\cdot\left( {a/r} \right)^{5} } \right],$$

where $a$ and r are protein hydrodynamic radius and pore radius, respectively. These values are reported in Table 1. Barrier permeability to IgG molecules can be described in the same way.

#### Evaluation of the sink term

Since protein efflux occurs by CSF drainage [23], the protein efflux pathways are the same as for CSF [28]. These include the arachnoid granulations mainly expressed in the cranial space but to a minor extent also in the spinal subarachnoid space, and outflow paths along nerves in both cranial and spinal spaces [29]. Thus, the drainage sink term can be written as

8 $$S_{o} = F\cdot C,$$

where *F* is the CSF drainage rate. The total CSF turn-over rate has been estimated to 500 ml/day in humans [30]. However, the distribution of the corresponding drainage between cranial and spinal compartments is not fully known [30], let alone its distribution along the spinal axis. To address this issue, we leverage available data on the spatial distribution of albumin concentrations at steady state, namely the known relative concentrations of albumin in the cisterns, lumbar and cortical subarachnoid spaces, and reported albumin concentration gradients along the spinal subarachnoid space.

At steady state, the average concentration in a given compartment can be derived from Eq. 5 and is established by the balance of the source and sink terms. Equating the source and sink terms given in Eqs. 6 and 8, we obtain the following expression for the albumin quotient, Q_Al_, in a given CSF compartment [6]:

9 $$Q_{Al} = \frac{{P_{bc} }}{{P_{bc} + \overline{{F_{c} }} }},$$

where the subscript c represents the CSF compartment for which Q_Al_ is known, namely the cisterns, cortical or spinal subarachnoid spaces, P_bc_ stands for the barrier permeability in that compartment and $\overline{{F_{c} }}$ for the mean CSF drainage rate to be determined. The corresponding results are reported in Table 3. The obtained mean drainage characteristics for the spinal compartment, $\overline{{F_{spinal} }}$, are then employed as baseline for other tested scenarios.

Having calculated the mean CSF drainage rate for the spinal compartment, we determine its local value by making use of reported albumin concentration gradients along the neuraxis. Due to the low CSF turnover rate, sequential sampling of CSF through a lumbar puncture allows one to sequentially access CSF portions from the lumbar, thoracic and finally cervical subarachnoid spaces. Using this method, a decrease of Q_Al_ was observed from the first 0–3 ml of CSF to the last 27–30 ml of CSF obtained by lumbar puncture [31]. Having an opposite gradient in CSF drainage has been hypothesized as the most probable mechanism for these changing CSF protein concentrations [6]. Accordingly, we assume spinal CSF drainage to increase linearly from zero at x = 0 in the lumbar sac (end of lumbar region) to twice $\overline{{F_{spinal} }}$ in the cervical region, thereby ensuring that the average spinal drainage matches the above determined value, $\overline{{F_{spinal} }}$. Note that only at exactly x = 0 is CSF drainage zero, but that integrated over a segment, for example along the lumbar region, there is CSF drainage.

#### Solution method

Equation (5) for solute transport is discretized using finite differences in Matlab with a forward Euler time stepping scheme and second order central differences for the spatial second derivatives. Neumann boundary conditions of zero flux for concentrations are imposed on the proximal end of the cervical region and the distal end of the lumbar space. These zero flux boundary conditions are reasonable due to the closed end of the lumbar and the steady-state equilibrium between protein influx and efflux in the lumped compartment of cranial space. The equation is solved with a time-step size of 6 s and a spatial resolution of 3.5 mm, with confirmed time-step and grid independence.

### The Reiber diagram

Reiber showed that a hyperbolic function can describe the relationship between albumin and immunoglobulin quotients seen in a population of patients without intrathecal production of immunoglobulins [32]:

10 $$Q_{IgG} = \frac{a}{b}\sqrt {Q_{Al}^{2} + b^{2} } - c,$$

where a, b and c are parameters appropriately chosen to fit the measured patient values. We use this empirical relationship as a reference for the output of the protein distribution.

Reiber further showed that the population variation coefficient, CV, stays constant as the albumin quotient changes. CV is defined as the ratio of the IgG variation to its mean value [32]:

11 $${\text{C}}V = \frac{{Q_{{IgG_{upper limit} }} - Q_{{IgG_{lower limit} }} }}{{0.5\cdot\left( {Q_{{IgG_{upper limit} }} + Q_{{IgG_{lower limit} }} } \right)}},$$

## Results

### Transport of the molecules in the spinal canal

We interrogated the 3D axisymmetric model to evaluate protein transport resulting from pulsatile spinal CSF motion. The diffusion coefficients of albumin and IgG in CSF are 6 · 10^−11^ and 2.4 · 10^−11^ m^2^/s, respectively. A peak CSF velocity of 10 mm/s was considered as Ref. [25]. The resulting dispersion coefficients are summarized in Table 2. Since the CSF pulsation amplitude reduces along the spinal canal towards the lumbar space [24], we also calculated the dispersion coefficient for lower velocities. Puy et al. showed that CSF pulsations can change in pathological situations [33], demonstrating an up to four fold increase in amplitude. To evaluate the impact of such pathological variations on protein distribution, we also calculated dispersion coefficients for accordingly increased velocities. We observed an almost linear increase in the dispersion coefficients with increasing velocity amplitude.

**Table 2:** Calculated protein dispersion coefficients

Molecule	Diffusion coefficient (m^2^/s)	Maximum CSF velocity (mm/s)	Dispersion coefficient (m^2^/s)
Immunoglobulin G	2.4 · 10^−11^	10	4.0 · 10^−8^
Albumin	6.0 · 10^−11^	2.5	2.8 · 10^−9^
		5	2.2 · 10^−8^
		10	6.0 · 10^−8^
		20	1.3 · 10^−7^
		40	2.7 · 10^−7^

### Distribution of albumin and IgG in the spinal CSF: baseline condition

**Fig. 4:**
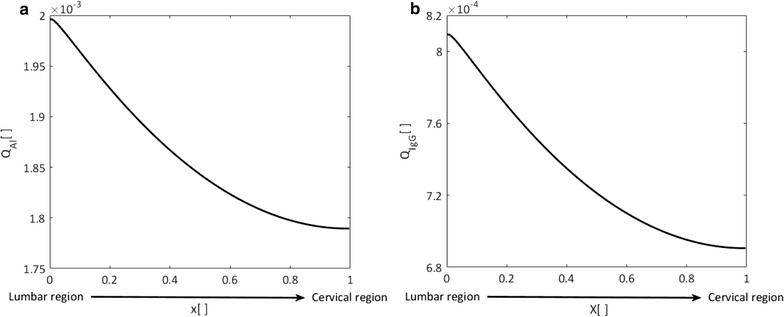
Albumin (**a**) and IgG (**b**) quotient distributions in the spinal cerebrospinal fluid. x is the normalized location on the rostro-caudal axis from lumbar (x = 0) to cervical space (x = 1) as illustrated in Fig. 3a. Quotients are obtained using spinal CSF drainage rates calculated as outlined in the “Methods” section. Permeability of the blood-CNS barrier to IgG is obtained using Eq. (7)

We first determined the distribution of CSF drainage between cortical and spinal spaces as outlined in the "Methods" section and then calculated albumin and IgG quotients using the one-dimensional model. Drainage distribution and albumin quotients in different regions of the CSF space are summarized in Table 3. The distribution of albumin and IgG quotients in the spinal canal between lumbar and cervical regions is shown in Fig. 4.

**Table 3:** CSF drainage distribution and albumin quotients in different CSF compartments

**CSF drainage distribution**	
Cortical region	82%
Spinal region	18%
**Albumin quotients in different CSF compartments**	
Lumbar region	0.002
Cortical subarachnoid space	0.003
Cistern	0.001

### Impact of CSF pulsation amplitude change on protein distribution

**Fig. 5:**
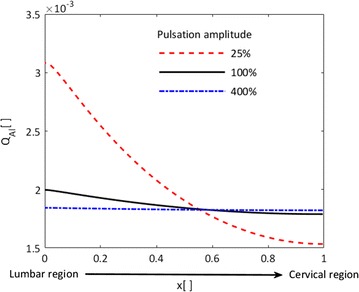
Impact of changes in CSF pulsation amplitude on the steady state albumin quotient distribution. x is the normalized location on the rostro-caudal spinal axis from lumbar (x = 0) to cervical space (x = 1) in Fig. 3a. The *solid black line* represents the nominal condition with CSF velocity pulsation amplitude of 10 mm/s (dispersion coefficient of 6 · 10^−8^ m^2^/s), the *red dashed* and *blue dashed*-*dotted lines* represent conditions with a factor of four pulsation amplitude reduction or increase, respectively (dispersion coefficients: 6 · 10^−8^ and 3.6 · 10^−8^ m^2^/s, respectively). Higher CSF velocity amplitudes reduce albumin gradients in the spinal cerebrospinal fluid space

We employed the 1D model of albumin distribution in conjunction with the dispersion rates obtained using the 3D model of protein transport in the spinal space to assess the effect of changes in CSF pulsation amplitude. We investigated the effect of a fourfold increase in CSF pulsation amplitude observed in chronic hydrocephalus patients [33] and used the corresponding dispersion coefficient calculated in the previous section. Figure 5 shows the impact of CSF pulsation amplitude change on the steady state albumin distribution in the spinal CSF. An increase in CSF velocity amplitude results in a more even albumin distribution in the spinal canal, whereas a decrease intensifies the concentration gradient.

### Impact of barrier dysfunction and CSF drainage on protein quotients

**Fig. 6:**
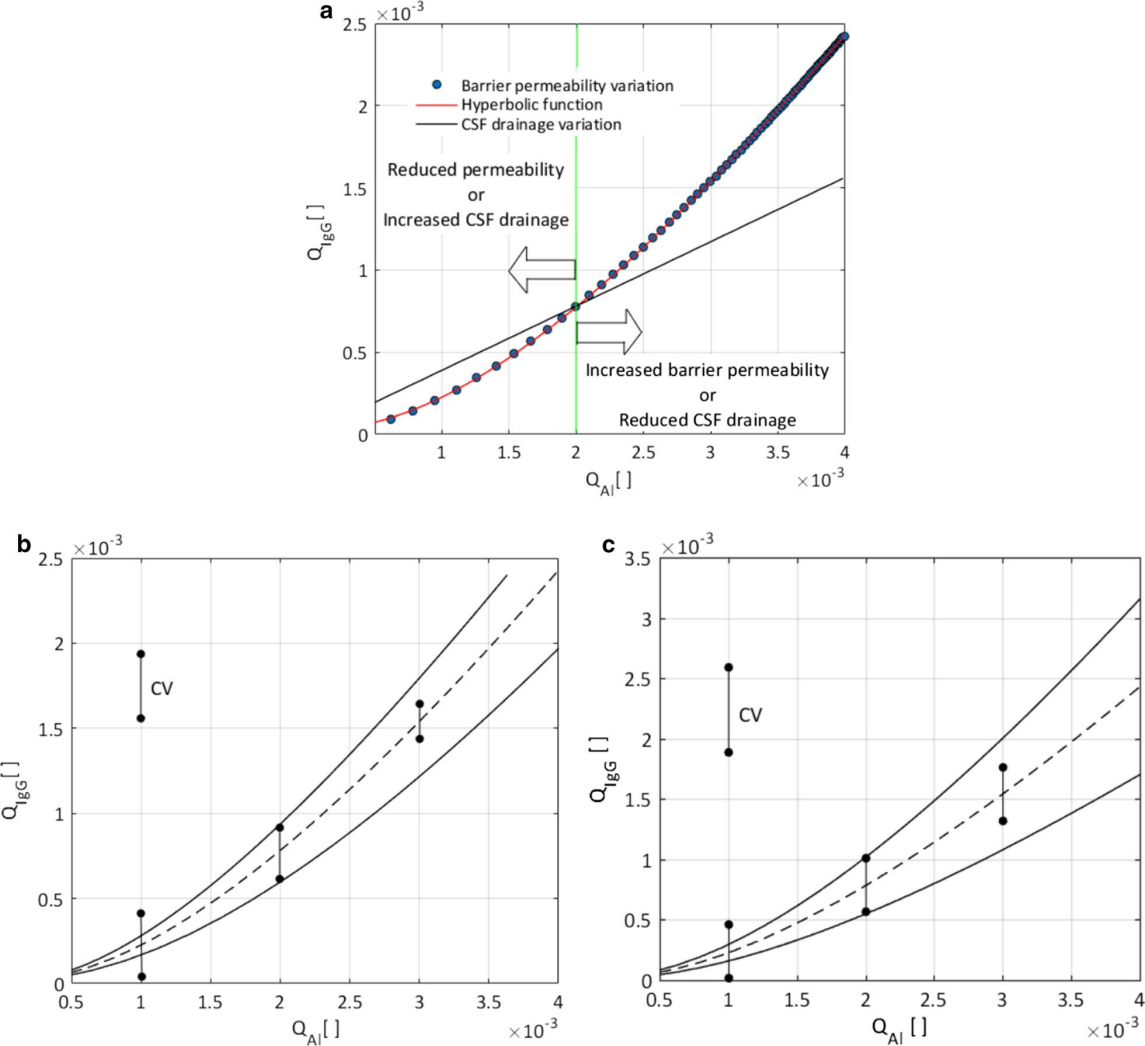
Relationship between IgG quotient and albumin quotient as commonly shown in the Reiber diagram. **a** *Blue circles* show how isolated changes in barrier permeability shape the relation between IgG and albumin quotients, while the *black solid line* demonstrates the corresponding effect of isolated changes in CSF drainage rate. The albumin quotient of 0.002 is taken as the nominal value. Decrease in CSF drainage and increase in barrier permeability lead to increased IgG and albumin quotients, and vice versa. Quotient variations due to changes in barrier permeability are perfectly described by the hyperbolic function (Eq. 10) empirically derived by Reiber (*red solid line*, R_square_ = 1). In contrast, quotient variations due to changes in CSF drainage follow a linear trend. **b** Quotient variation due to barrier permeability change. The *dashed line* represents nominal CSF drainage conditions, while the *upper* and *lower solid lines* are representative of 30% increased and decreased CSF drainage rates, respectively. The population variation coefficient for albumin quotients of 0.001, 0.002, 0.003 is, respectively, 0.48, 0.44 and 0.4. **c** The effect of barrier permeability change for three different baseline IgG permeabilities. The *dashed line* represents the nominal IgG permeability and *upper and lower solid lines* represent 30% increased and decreased IgG baseline permeability, respectively. The calculated variation coefficient is constant (with a value of 0.6) for all albumin quotients

We used the 1D model to investigate the effect of changes in blood-CNS barrier permeability and CSF drainage on albumin and IgG quotients in the lumbar cerebrospinal fluid. Figure 6a shows the relationship between IgG and albumin quotients in the cases of barrier permeability change (circles) and CSF drainage rate change (solid black line). An albumin quotient of 0.002 is taken as the nominal value. Decrease in CSF drainage and increase in barrier permeability lead to increased IgG and albumin quotients, and vice versa. The empirical hyperbolic relation between albumin and IgG quotients derived by Reiber [2] from measurements in patients’ CSF samples is shown to match well with our calculations for barrier permeability change (solid red line).

Figure 6b illustrates the effect of change in barrier permeability for three different constant CSF drainage rates. The center (dashed) curve corresponds to nominal drainage, while the upper and lower solid curves correspond to 30% increased and decreased drainage rates, respectively. All three curves are hyperbolic. We used the upper and lower curves to calculate representations of the population variation coefficient, obtaining values of 0.48, 0.44 and 0.4 for albumin quotients of 0.001, 0.002 and 0.003, respectively. Note that the population variation coefficient determined by Reiber based on patient data is constant over a range of albumin quotients.

Figure 6c illustrates the effect of change in barrier permeability for three different baseline IgG permeabilities, reflecting the variation of the barrier permeability to IgG to different extent than for albumin as shown by Seyfert et al. [34]. The center (dashed) curve corresponds to nominal baseline IgG permeability, while the upper and lower solid curves correspond to 30% increased and decreased baseline IgG permeability, respectively. The representation of the population variation coefficient is in this case 0.6 for all albumin quotients.

## Discussion

The biochemical analysis of the cerebrospinal fluid is an important diagnostic tool for pathologies of the CNS. For example, changes in CSF immunoglobulin content can be indicative of inflammatory reactions in the brain. To account for inter-individual and normal intra-individual variability, it is advantageous to assess relative rather than absolute values of protein concentration as done in the Reiber diagram. While the Reiber diagram can indicate intrathecal synthesis of proteins, it is debated whether higher than normal readings of relative albumin concentrations are indicative of CNS barrier dysfunction or reduction in CSF drainage. Here we have employed a set of computational models to assess which one of these two changes is the more likely cause of increased albumin concentration in CSF relative to that in the blood plasma.

The Reiber diagram features a hyperbolic relationship between albumin quotient and, for example, IgG quotient, where ‘quotient’ refers to the concentration of the respective protein in CSF relative to its concentration in blood plasma. Reiber derived this empirical relationship from measurements in a large set of patients in which intrathecal synthesis of the protein of interest could be excluded. He hypothesized that this non-linear relationship was caused by inter-patient variability in CSF drainage rates [32]. However, as shown in Fig. 6a, our models indicate that variations in the rate of CSF drainage would yield a linear relationship between the quotients rather than the experimentally determined hyperbolic one. Reiber also calculated the variation coefficient for his patient database and found it to be constant for a large range of albumin quotients. Our calculations show that the variation coefficient does not stay constant for different baseline CSF drainage values (Fig. 6b), indicating that inter-patient variability in CSF drainage alone may not result in the protein quotient relationship observed by Reiber. One should thus not, without further case-dependent evidence, attribute abnormally high albumin quotients identified in the Reiber diagram to reduced CSF drainage.

Others have attributed increased albumin quotients to blood-CNS barrier dysfunction. Indeed, as shown in Fig. 6a, variation in barrier permeability leads to the expected hyperbolic relationship between protein quotients. This is further confirmed by a constant population variation coefficient as illustrated in panel (c) for different baseline IgG permeabilities. Consequently, high albumin quotients identified in the Reiber diagram may be seen as indicative of a CNS barrier dysfunction.

Our calculations of the distribution of CSF efflux indicate 18% drainage in the spinal compartment and 82% drainage in the cranial compartment. This distribution matches well with the measurements of Marmarou et al. [35] in cats, where absorption in the spinal space accounted for 16% of the total CSF drainage and the cranial space contributed 84%. Similar results were obtained by Gehlen et al. using a lumped parameter model of coupled cardiovascular and CSF dynamics [36]. Albumin quotients calculated based on this drainage distribution are within the range of values obtained experimentally in healthy subjects [31].

Seyfert et al. measured albumin and immunoglobulin concentration gradients in the spinal CSF by sequential CSF sampling through lumbar puncture. They showed a decreasing protein concentration profile from lumbar to cervical space [31]. It was hypothesized that this concentration gradient results from the variation of CSF drainage along the spine [6]. Our calculations show that the hypothesized drainage gradient along the spinal canal with minimum drainage rate in the lumbar space would, indeed, result in a longitudinal concentration gradient for albumin and IgG (Fig. 4). Therefore, our results support the existence of rostro-caudally decreasing spinal CSF drainage.

Puy et al. correlated the magnitude of CSF pulsation with protein distribution in different CSF compartments [33]. We calculated the dispersion rate of albumin in the spinal CSF for different pulsation amplitudes as reported in Table 2, and employed these values in our global protein distribution model. Increased CSF pulsation diminishes the longitudinal concentration gradient in the spinal canal, while reduced pulsation intensifies it (Fig. 5). These results are in line with the measurements of Puy et al. [33]. Therefore, changed CSF dynamics in pathologies such as hydrocephalus and Chiari malformation could have an impact on protein distribution in the spinal canal.

The two computational models developed in this study have the following main limitations: First and foremost, we have simplified the spinal canal anatomy substantially to a 3D axisymmetric annular conduit and a 1D representation, respectively, considering the spinal subarachnoid space as a porous medium. Both the macroscopic anatomy as well as the microanatomy of the CSF spaces as defined by, e.g. arachnoid trabeculae, could play an important role in fluid and solute dynamics. Neglecting the microanatomy can lead to discrepancies between computed and measured metrics of spinal CSF dynamics [19]. In our models, the effect of microstructures is approximated by the introduction of anisotropic permeability of the porous medium representing the spinal subarachnoid space.

The second main limitation pertains to the issue of parameter uncertainty. For instance, we have considered the overall CSF drainage rate to be equal to the estimated value of CSF production, which itself is only known approximatively [30]. We have dealt with parameter uncertainty by performing sensitivity analyses, which show that our main conclusions are robust with respect to reasonable variations of the model parameters. Concretely, we have shown that the hyperbolic protein quotient function in the Reiber diagram that results from variation in barrier permeability does not depend on baseline CSF drainage (Fig. 6b) or IgG permeability values (Fig. 6c). We have also made sure that the population variation coefficient does not only stay constant for a 30% change in IgG baseline permeability (Fig. 6c), but also for much larger and smaller changes (up to 100% change). Finally, we checked that the derived dispersion coefficients do not depend on the computational domain length and hydraulic conductivity of the domain.
